# Confocal laser endomicroscopy in breast surgery: a pilot study

**DOI:** 10.1186/s12885-015-1245-6

**Published:** 2015-04-10

**Authors:** Giovanni D De Palma, Dario Esposito, Gaetano Luglio, Gennaro Limite, Antonello Accurso, Viviana Sollazzo, Francesco Maione, Gianluca Cassese, Saverio Siciliano, Nicola Gennarelli, Gennaro Ilardi, Mariano Paternoster, Mariano C Giglio, Pietro Forestieri

**Affiliations:** 1 Department of Clinical Medicine and Surgery, University of Naples Federico II. School of Medicine, Naples, Italy; 2 Department of Advanced Biomedical Sciences, University of Naples Federico II. School of Medicine, Naples, Italy

## Abstract

**Background:**

Breast neoplasms include different histopathological entities, varying from benign tumors to highly aggressive cancers. Despite the key role of imaging, traditional histology is still required for a definitive diagnosis. Confocal Laser Endomicroscopy (CLE) is a new technique, which enables to obtain histopathological images in vivo, currently used in the diagnosis of gastrointestinal diseases. This is a single-center pilot feasibility study; the main aim is to describe the basic morphological patterns of Confocal Laser Endomicroscopy in normal breast tissue besides benign and malignant lesions.

**Methods:**

Thirteen female patients (mean age 52.7, range from 22 to 86) who underwent surgical resection for a palpable breast nodule were enrolled. CLE was performed soon after resection with the Cellvizio® Endomicroscopy System (Mauna Kea Technologies, Paris, France), by using a Coloflex UHD-type probe; intravenous fluorescein was used as contrast-enhancing agent. The surgical specimen was cut along the main axis; dynamic images were obtained and recorded using a hand-held probe directly applied both to the internal part of the lesion and to several areas of surrounding normal tissue. Each specimen was then sent for definitive histologic examination.

**Results:**

Histopathology revealed a benign lesion in six patients (46%), while a breast cancer was diagnosed in seven women (54%). Confocal laser endomicroscopy showed some peculiar morphological patterns. Normal breast tissue was characterized by a honeycomb appearance with regular, dark, round or hexagonal glandular lobules on a bright stroma background; tubular structures, representing ducts or blood vessels, were also visible in some frames. Benign lesions were characterized by a well-demarcated “slit-like” structure or by lobular structures in abundant bright stroma. Finally, breast cancer was characterized by a complete architectural subversion: ductal carcinoma was characterized by ill-defined structures, with dark borders and irregular ductal shape, formingribbons, tubules or nests; mucinous carcinoma showed smaller cells organized in clusters, floating in an amorphous extracellular matrix.

**Conclusions:**

This is the first pilot study to investigate the potential role of confocal laser imaging as a diagnostic tool in breast diseases. Further studies are required to validate these results and establish the clinical impact of this technique.

## Background

Breast neoplasms include different histopathological entities, varying from benign tumors to highly aggressive cancers. Clinical aspects of breast lesions include nodules, lumps or cysts; the possibility to differentiate between benign and malignant lesions is a key factor in order to establish the proper management protocol and determine whether surgery is needed. Mammography, Ultrasonography and even MRI are commonly used in breast diseases work-up; however, despite the accuracy of modern imaging, traditional histology is still required for a definitive diagnosis.

Ultrasonography, X-ray and MRI are used for targeted biopsies, but excisional biopsy is often required nonetheless.

Confocal Laser Endomicroscopy (CLE) is a relatively new technique that enables real-time histopathology of human tissues. Confocal microscopy is based on focusing a laser beam (such as an argon-ion laser that generates an excitation wavelength of 488 nm, blue laser light) into a plane of interest; the returning light is filtered through a small pinhole that rejects the out-of-focus light. Illumination and detection systems operate in the same focal plane and are referred as “confocal”. After passing the pinhole, the fluorescent light is detected by a photo-detection device that transforms the light signal into an electrical one that is recorded by a computer system. All signals coming from illuminated spots are captured and measured. Laser scanning over a plane of interest allows to obtain a pixel-by-pixel and line-by-line image, whereas the brightness of each single pixel corresponds to the relative intensity of the detected fluorescent light. A gray scale image is created which corresponds to an optical section and represents the focal plane within the examined specimen. A fluorescent dye (contrast agent) is required to make confocal images visible: contrast agents can be administered systemically (fluorescein, tetracycline) or topically (acriflavine, cresyl violet), using a spraying catheter. Intravenous fluoresceinsodium (10%) and topically applied acriflavine (0.2%) have been most commonly used in humans [1,2].

CLE has been first applied in the field of gastrointestinal endoscopy, showing to be a very useful tool in targeted biopsies, thus reducing the number of samples and increasing the diagnostic accuracy, especially in clinical settings such as Barrett’s Esophagus, Ulcerative Colitis and early identification of dysplasia [3-6]. Recently, applications in gastrointestinal surgery, pulmonology and neurosurgery have also been described [7-10].

The aim of the present study was to describe the basic morphological patterns at CLE in normal breast tissue and in benign and malignant lesions; this might represent the first step for further investigation into assessing the role of CLE as a diagnostic tool in breast diseases.

## Methods

### General study design

This is a prospective, single-center, pilot-study; the primary aim was to describe typical morphological patterns in normal breast tissue and in benign and malignant lesions at CLE (confocal laser endomicroscopy). The feasibility of CLE combined with video-mosaicing was also investigated, together with its ability to differentiate between normal, benign and malignant breast tissue. The secondary aim was to investigate the ideal timing for fluoresceine i.v. infusion before specimen resection, when fluoresceine is used as contrast-enhancing agent. The study was approved by the internal institutional review board “Carlo Romano” (protocol n. 276/2013). A signed informed consent was obtained from all the patients enrolled in the study.

### Patients

All patients, as from inclusion criteria, were older than 18 and had breast lesion requiring surgical excision. Exclusion criteria were: pregnancy, allergy to fluorescein or drugs, chronic renal failure, inability to understand the study protocol.

### Equipment

CLE was performed by using the Cellvizio® Endomicroscopy System (Mauna Kea Technologies, Paris, France) with a Coloflex UHD-type probe (1 μm lateral resolution; 12 frames/s). This probe has a 240 μm × 200 μm field of view, with a lateral resolution of 1 μm. CLE imaging data were collected at a 12 frames/s scan rate, the scanning field being of 30 000 pixels.

### Tissue examination

All the patients with malignant lesions were treated with quadrantectomy; a simple excision was performed in case of benign nodules. During surgery, patients received 5 ml of intravenous 10% fluorescein, about 5 minutes before the specimen excision (time range: 2 to 10 minutes).

The surgical specimen was examined by CLE immediately after the resection. The specimen was cut along the main axis and dynamic images were collected by using the hand-held probe, applied directly both to the internal part of the lesion and to several areas of surrounding normal tissue. Care was taken while examining the margins of the lesion in order to recognize the transition between normal and neoplastic tissue.

Confocal laser imaging was carefully analyzed in order to describe cellular morphology and cellular structures of specimens. Differences between macroscopically normal or neoplastic tissue were evaluated in real time, in order to describe peculiar morphological patterns attributing them to normal tissue, benign or malignant lesions.

Proper timing for i.v.fluorescein infusion has also been assessed, in order to establish the best interval from fluorescein infusion to specimen resection. All the images and mosaics were stored in the Cellvizio® Endomicroscopy System database. Specimens were then sent for traditional histology.

## Results

Thirteen female patients were enrolled with a mean age of 52.7 (range 22 – 86). A fibroepithelial lesion was diagnosed in six patients (46%): 4 of them had a fibroadenoma and 2 had a benign phyllodes tumor. An invasive breast cancer was diagnosed in seven patients (54%): five patients had a ductal carcinoma, one patient had a lobular carcinoma and one patient a mucinous carcinoma (Table 1).

**Table 1:** **Patients characteristics**

N.	Age	Histology	Receptor status°	Grade (G)*	Stage (TN)
1	22	Phylloid			
2	55	Ductal Carcinoma	ER 90%, PR 70%, Ki-67 15%, Her-2 neg	G2	pT1, N0
3	74	Ductal Carcinoma	ER 90%, PR 90%, Ki-67 5%, Her-2 3+	G3	pT1, Nx
4	30	Fibroadenoma			
5	77	Ductal Carcinoma	ER 90%, PR 2%, Ki-67 15%, Her-2 neg	G3	pT2, N1
6	29	Fibroadenoma			
7	64	Lobular Carcinoma	ER 80%, PR 0%, Ki-67 5%, Her-2 neg	G2	pT2, N1
8	19	Fibroadenoma			
9	42	Fibroadenoma			
10	42	Phylloid			
11	75	Ductal Carcinoma	ER 90%, PR 70%, Ki-67 25%, Her-2 neg	G3	pT1, N0
12	86	Mucinous Carcinoma			
13	71	Ductal Carcinoma	ER 90%, PR 2%, Ki-67 15%, Her-2 neg	G3	pT2, N1

°ER (estrogen receptor); PR (progesterone receptor) Her-2 (Human epidermal growth factor).

*Tumor Grading system: G1: well differentiated; G2: moderately differentiated; G3: poorly differentiated; G4: undifferentiated.

### Morphological pattern at confocal laser imaging

**Figure 1:**
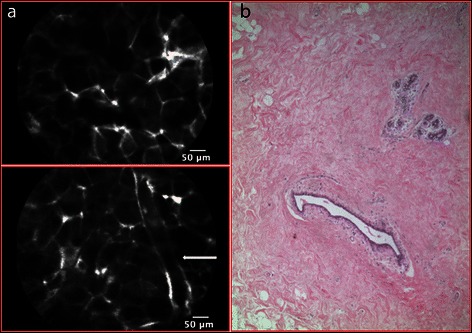
p-CLE imaging of normal breast. **a**: CLE-fluorescein sodium 10% imaging of normal breast tissue characterized by a honeycomb appearance with regular dark round or hexagonal glandular lobules in a bright stroma background. A tubular structure representing a duct is visible in this frame (arrow). **b**: Corresponding normal breast tissue (haematoxylin and eosin staining).

Normal breast tissue was characterized by a honeycomb appearance, with regular, dark, round or hexagonal glandular lobules on a bright stroma background; in some frames there were also tubular structures, representing ducts or blood vessels (Figure 1).

**Figure 2:**
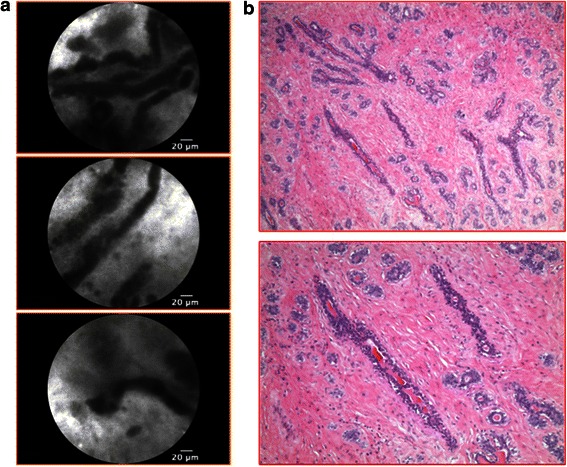
p-CLE imaging of intracanalicular fibroadenoma. **a**: CLE-fluorescein sodium 10% imaging showing well-demarcated “slit-like” structures in an abundant bright stroma, representing irregular and compressed ducts. **b**: Corresponding histopathological image (haematoxylin and eosin staining).

**Figure 3:**
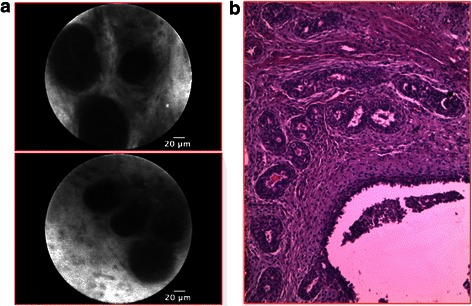
p-CLE imaging of pericanalicular fibroadenoma. **a**: CLE- fluorescein sodium 10% imaging showing round or oval structures representing ductal spaces in an abundant bright stroma. **b**: Corresponding histopathological image (haematoxylin and eosin staining).

Fibroepithelial lesions were characterized by well-demarcated, “slit-like”, lobular and darker structures in a bright stroma; nuclei of fibroblast cells were also clearly visible in some frames. “Slit-like” figures might be the expression of compressed and irregular ducts typical of *intracanalicular* fibroadenoma (Figure 2); on the other hand, “lobular structures” are typical of *pericanalicular* fibroadenoma, with a morphological pattern similar to the one seen at traditional histology (Figure 3).

**Figure 4:**
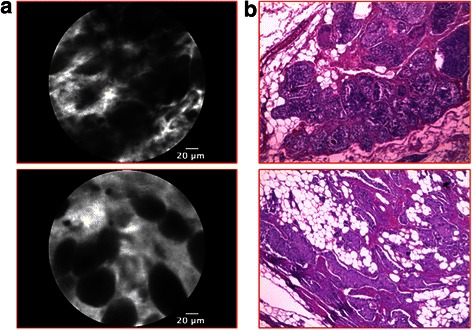
p-CLE imaging of infiltrating ductal carcinoma. **a**: CLE- fluorescein sodium 10% imaging showing a complete architectural subversion, with ill-defined structures, dark border and irregular ductal shape forming ribbons, tubules or nests. **b**: Corresponding histopathological image (haematoxylin and eosin staining).

**Figure 5:**
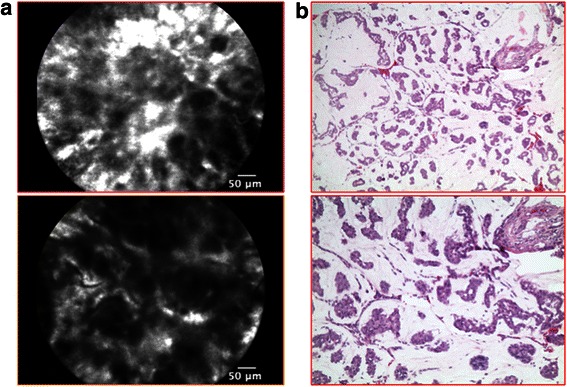
p-CLE imaging of invasive mucinous carcinoma. **a**: CLE- fluorescein sodium 10% imaging showing smaller cells organized in clusters, floating in an amorphous extracellular mucin matrix**. b**: Corresponding histopathological image (haematoxylin and eosin staining).

Malignancies were characterized by a complete architectural subversion. *Ductal carcinoma* showed ill-defined structures with dark borders and irregular ductal shape, forming ribbons, tubules or nests. Single, small infiltrating cells were also present, thus forming a hypercellular stroma (Figure 4). *Mucinous carcinoma* had similar features with smaller cells organized in clusters and floating in an amorphous background of extracellular mucin (Figure 5).

We exclusively used fluoresceine as contrast-enhancing agent; the major drawback of this agent is its difficulty in highlighting cell nuclei, thus it was not possible to evaluate the presence of mitotic figures and proliferation rate. Acriflavine is a different contrast-enhancing agent that is able to penetrate into cell nuclei and might be useful to identify mitotic figures; unfortunately its use in-vivo is limited by a mutagenic potential.

**Figure 6:**
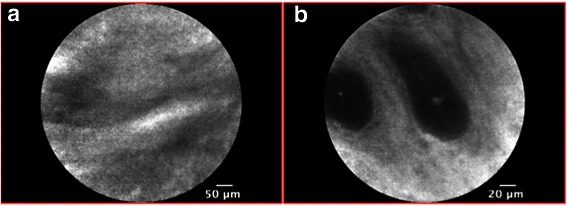
Evaluation of image quality with increasing injection-to-imaging time in benign breast lesions. Figure shows the poor image quality after a “short” infusion interval (**a**: 2 min) and how it gets better when a longer (**b**: 5 min) interval has been used.

We initially used a short interval (2 minutes) between fluorescein administration and specimen resection, which we think resulted in poor image quality, especially for benign nodules; this was probably due to the hypovascularization of such lesions. A five-minute interval seemed to be required for optimal fluoresce in impregnation of benign lesions. Figure 6 shows the poor image quality after a “short” infusion interval and how the image-quality gets better when a longer (5 min) interval has been used. No significant differences in image quality was observed in malignant lesions relating to different timing of infusion.

## Discussion

Diagnosis of breast diseases is usually based on radiology and traditional histopathology. The four-parameter 18-FDG-PET/MRI has high accuracy in differential diagnosis between benign and malignant neoplasms of breast, leading to a reduction of unnecessary biopsies [11].

The present pilot study showed, for the first time, that CLE might be a useful tool in highlighting morphological features of normal breast, benign and malignant lesions, thus anticipating histopathological diagnosis.

In this preliminary experience, benign lesions seemed to require a longer interval from fluorescein i.v. infusion to CLE analysis, compared with the malignant ones; this finding may need further confirmation in larger sample studies. Moreover, a different timing in fluorescein tissue impregnation might be also used as an a adjunctive diagnostic criterion, in discriminating benign from malignant lesions. We used fluorescein as contrast-enhancing agent, which is the most common agent used to date. However different fluorescent dyes are available (e.g. acriflavine) that might be applied topically to highlight cellular nuclei. Furthermore, different light sources like NIR (near infrared probes) or dual wavelength light sources could be used in the future to increase penetration depth of scanning light [12,13].

Further investigation in this field might lead to routine use of in-vivo US-guided or radio-guided virtual biopsies; new “needle probes” might be also required, similar to the ones already in use in the field of US-Endoscopy [14,15].

Real time, in-vivo, virtual histology could significantly decrease the time needed for a diagnosis or, at least, provide a useful tool in discriminating lesions that will require further histological assessment.

An additional clinical use of virtual histology might be the assessment of resection margins, immediately after the surgical resection or even directly in the operating field, thus saving time when extemporary histology would be required anyway. Something similar has been already proposed in colorectal and neurosurgery [7,10].

Despite CLE produces “microscopy” images, it does not generally require an expert pathologist interpretation; the role of CLE has already been established in the field of gastrointestinal endoscopy, where it has been demonstrated to be a useful tool for surgeons or endoscopists in targeted biopsies. Our experience demonstrates similar findings; we believe that a training together with a dedicated pathologist would be sufficient to learn how to discriminate the main morphological patterns in order to differentiate between malignant and benign lesions. On the other hand, the role of CLE in the field of breast cancer needs to be further investigated. One might argue that once the skin is opened, CLE might not give any real advantages compared to traditional histology with regard to invasiveness. We actually see a potential role of this investigation in those situations where an extemporaneous examination is required: CLE is a time-saving examination, does not require sample preparation or staining and might allow the surgeon to achieve a diagnostic orientation immediately at the operating table.

## Conclusions

This is a pilot study to investigate the potential role of confocal laser imaging as diagnostic tool in breast diseases. Different morphological patterns at CLE examination have been carefully described, both in benign and malignant breast lesions. Further studies are needed to validate these results and establish the clinical impact of this technique.

## Abbreviations

MRI: Magnetic resonance imaging
CLE: Confocal laser endomicroscopy
18-FDG-PET/MRI: 18fluoro-2-d-glucose positronemissiontomography with computedtomography
NIR: Near infrared probes

## References

[1] De Palma GD (2009). Confocal laser endomicroscopy in the “in vivo” histological diagnosis of the gastrointestinal tract. World J Gastroenterol.

[2] De Palma GD, Wallace MB, Giovannini M (2012). Confocal laser endomicroscopy. Gastroenterol Res Pract.

[3] De Palma GD, Staibano S, Siciliano S, Maione F, Siano M, Esposito D (2011). In-vivo characterization of DALM in ulcerative colitis with high-resolution probe-based confocal laser endomicroscopy. World J Gastroenterol.

[4] Rispo A, Castiglione F, Staibano S, Esposito D, Maione F, Siano M (2012). Diagnostic accuracy of confocal laser endomicroscopy in diagnosing dysplasia in patients affected by long-standing ulcerative colitis. World J Gastrointest Endosc.

[5] Mascolo M, Staibano S, Ilardi G, Siano M, Vecchione ML, Esposito D (2012). Probe-based confocal laser endomicroscopy evaluation of colon preneoplastic lesions, with particular attention to the aberrant crypt foci, and comparative assessment with histological features obtained by conventional endoscopy. Gastroenterol Res Pract.

[6] Dunbar KB, Okolo P, Montgomery E, Canto MI (2006). Confocal laser endomicroscopy in Barrett’s esophagus and endoscopically inapparent Barrett’s neoplasia: a prospective, randomized, double-blind, controlled, crossover trial. Gastrointest Endosc.

[7] De Palma GD, Luglio G, Staibano S, Bucci L, Esposito D, Maione F (2014). Perioperative characterization of anastomotic doughnuts with high-resolution probe-based confocal laser endomicroscopy in colorectal cancer surgery: a feasibility study. Surg Endosc.

[8] Fuchs FS, Zirlik S, Hildner K, Schubert J, Vieth M, Neurath MF (2013). Confocal laser endomicroscopy for diagnosing lung cancer in vivo. Eur Respir J.

[9] Chen SP, Liao JC (2014). Confocal laser endomicroscopy of bladder and upper tract urothelial carcinoma: a new era of optical diagnosis?. Curr Urol Rep.

[10] Foersch S, Heimann A, Ayyad A, Spoden GA, Florin L, Mpoukouvalas K (2012). Confocal laser endomicroscopy for diagnosis and histomorphologic imaging of brain tumors in vivo. PLoS One.

[11] Pinker K, Bogner W, Baltzer P, Karanikas G, Magometschnigg H, Brader P (2014). Improved differentiation of benign and malignant breast tumors with multiparametric 18fluorodeoxyglucose positron emission tomography magnetic resonance imaging: a feasibility study. Clin Cancer Res.

[12] Goetz M, Deris I, Vieth M, Murr E, Hoffman A, Delaney P (2010). Near-infrared confocal imaging during mini-laparoscopy: a novel rigid endomicroscope with increased imaging plane depth. J Hepatol.

[13] Jean F, Bourg-Heckly G, Viellerobe B (2007). Fibered confocal spectroscopy and multicolor imaging system for in vivo fluorescence analysis. Opt Express.

[14] Konda VJ, Meining A, Jamil LH, Giovannini M, Hwang JH, Wallace MB (2013). A pilot study of in vivo identification of pancreatic cystic neoplasms with needle-based confocal laser endomicroscopy under endosonographic guidance. Endoscopy.

[15] Giovannini M, Caillol F, Poizat F, Bories E, Pesenti C, Monges G (2012). Feasibility of intratumoral confocal microscopy under endoscopic ultrasound guidance. Endosc Ultrasound.

